# Beyond breastfeeding: a One Health Decalogue for nurturing the infant microbiota

**DOI:** 10.3389/fnut.2026.1784544

**Published:** 2026-03-05

**Authors:** Valentina Biagioli, Mariarosaria Matera, Ilaria Cavecchia, Mariateresa Illiceto, Laura Pennazzi, Gaia Luongo, Sebastian Lugli, Pasquale Striano

**Affiliations:** 1 Department of Neurosciences, Rehabilitation, Ophthalmology, Genetics, Maternal and Child Health, University of Genoa, Genoa, Italy; 2 Microbiota International Clinical Society, Turin, Italy; 3 Department of Pediatric Emergencies, Misericordia Hospital, Grosseto, Italy; 4 Microbiomic Department, Koelliker Hospital, Turin, Italy; 5 Department of Pediatrics-Unit of Pediatric Gastroenterology and Endoscopy, Santo Spirito Hospital, Pescara, Italy; 6 I.P.S.I.A. Attilio Odero – State Professional Technical Institute, Genoa, Italy; 7 Pediatric Neurology and Muscular Diseases Unit, IRCCS Istituto “Giannina Gaslini”, Genoa, Italy

**Keywords:** breastfeeding, complementary feeding, early-life microbiome, environmental exposures, microbiota

## Abstract

**Background:**

Early-life nutrition is a key determinant of infant gut microbiota development, immune maturation, and long-term health outcomes. Although breastfeeding is widely recognized as the optimal feeding strategy, many mothers are unable to breastfeed, underscoring the need for practical, evidence-based guidance to support infant health beyond breastfeeding. A One Health approach enables the integration of nutritional, microbial, clinical, environmental, and socio-cultural factors that influence maternal–infant dyads.

**Methods:**

A narrative review of the literature was conducted using PubMed, Scopus, and Google Scholar, focusing more on works published from 2020 to 2026. Evidence was synthesized on maternal and infant nutrition, breast milk bioactive components, infant formula feeding, gut microbiota development, and short- and long-term health outcomes in non-breastfed infants. Based on this interdisciplinary evidence, a translational “One Health Decalogue” was developed for mothers who are unable to breastfeed.

**Findings:**

The reviewed literature highlights that infant nutrition, particularly in the absence of breastfeeding, significantly influences gut microbiota composition, immune programming, metabolic regulation, and neurodevelopment. Key modifiable factors include formula composition, feeding practices, maternal health status, environmental exposures, caregiver education, and psychosocial support. The proposed One Health Decalogue synthesizes these elements into 10 actionable principles aimed at supporting microbial resilience, promoting healthy development, and reducing health inequalities when breastfeeding is not possible.

**Conclusion:**

Translating scientific evidence into practical tools is essential to support infants who cannot be breastfed. The One Health Decalogue presented in this review provides a comprehensive, interdisciplinary, and translational framework for healthcare professionals, families, and public health policies, fostering informed nutritional choices and holistic strategies to optimize infant health beyond breastfeeding.

## 1 Introduction

The World Health Organization (WHO) and the United Nations International Children’s Emergency Fund (UNICEF) recommend breastfeeding as the gold standard for infant nutrition. Beyond providing balanced macro- and micronutrients, human milk also contains bioactive compounds that support gut maturation, immune development, and neurological development (1).

However, according to UNICEF and WHO data, only 48% of infants are exclusively breastfed in the first 6 months of life. Breastfeeding has certainly increased compared to the past, but it is still below the 50% target set by the World Health Assembly for 2025. Epidemiological studies report that 20–30% of infants globally receive formula or mixed milk from the first days of life, with marked differences between high and low-income countries (2). The causes of failure to initiate or early cessation of breastfeeding are multifactorial and include maternal or neonatal pathologies, prematurity, and lactation difficulties, as well as cultural, socioeconomic, and employment barriers, and barriers to accessing health and social support. In particular, the lack of supportive practical guidelines and adequate parental leave policies, poor training of healthcare workers, and social pressures can significantly reduce the duration and quality of breastfeeding (2).

However, the first 1,000 days of life represent a critical, unique, and unrepeatable window in which the structure and maturation of the gut microbiota have lasting effects on immunological, metabolic, and neurobehavioral development and, therefore, on the individual’s long-term health (3–5). During this period, nutrition and environmental exposure interact in a complex and decisive way. Therefore, when exclusive breastfeeding is not possible, the composition of the infant’s diet (formula or mixed feeding, timing, and characteristics of weaning) and any interventions to modulate the microbiota play a crucial role in shaping the trajectory of microbial development (6, 7). For infants who cannot be breastfed, there is marked geographic, socioeconomic, and cultural variability in feeding practices, food availability, and even access to support and counseling. Many settings lack practical and integrated guidelines that consider not only basic nutrition, but also microbiota modulation and mother-infant health promotion (8, 9). This gap represents a critical public health issue.

**Figure 1:**
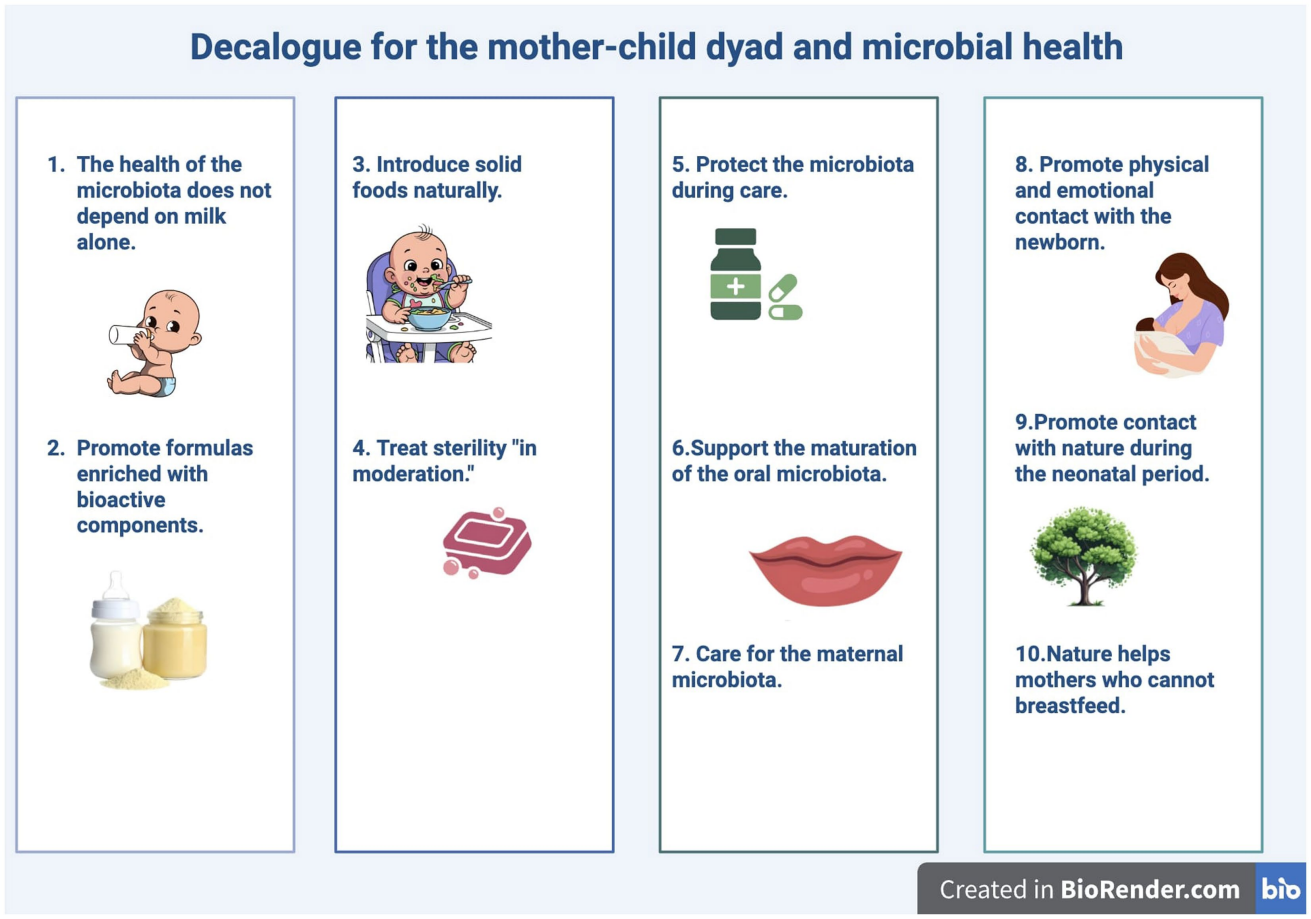
Summarized the decalogue points about the mother–child dyad and microbial health.

This review aims to propose an integrated approach, inspired by the principles of One Health and microbiome modulation, aimed at defining a practical and operational, evidence-based standard of care that can mitigate the risks associated with infant formula and formula-based formula. The ultimate goal is to contribute to the development of practical, translational, and interdisciplinary guidelines for healthcare professionals, families, and public health policies. To translate scientific evidence into operational tools, we have developed a “One Health Decalogue” for mothers who cannot breastfeed, which will be developed and commented on in detail in the subsequent sections of the review. The Decalogue is divided into the points summarized in Figure 1.

## 2 Materials and methods

### 2.1 Search strategy

A comprehensive narrative literature review was conducted to identify peer-reviewed studies addressing maternal and infant nutrition, breast milk composition, infant formula feeding, gut microbiota development, and health outcomes during early life, with particular focus on infants who are not exclusively breastfed. The search was performed using PubMed, Scopus, and Google Scholar databases, including articles published up to 2020.

Search terms were combined using Boolean operators and included: *“breastfeeding,” “infant formula,” “mixed feeding,” “infant nutrition,” “gut microbiota,” “early-life microbiome,” “human milk bioactive components,” “complementary feeding,” “environmental exposures,” “microplastics,” “endocrine disruptors,” “mother-infant dyad,”* and *“One Health.”* Additional manual searches were conducted by screening reference lists of relevant reviews, international guidelines, and policy documents from organizations such as WHO and UNICEF to ensure comprehensive coverage of clinical, microbiological, and public health perspectives.

### 2.2 Inclusion and exclusion criteria

Inclusion criteria comprised human studies involving neonates, infants, and mothers during the first 1,000 days of life; studies evaluating breastfeeding, formula feeding, or mixed feeding and their effects on gut microbiota, immune development, metabolic and neurodevelopmental outcomes; clinical trials, observational studies, cohort studies, and cross-sectional studies; systematic reviews and meta-analyses addressing infant nutrition and microbiota modulation; and relevant preclinical studies elucidating mechanistic links between early nutrition, microbial colonization, and health development.

Exclusion criteria included studies exclusively focused on adult populations; articles lacking relevance to early-life nutrition or gut microbiota development; papers with insufficient methodological rigor; and non-English publications without an available translation.

**Figure 2:**
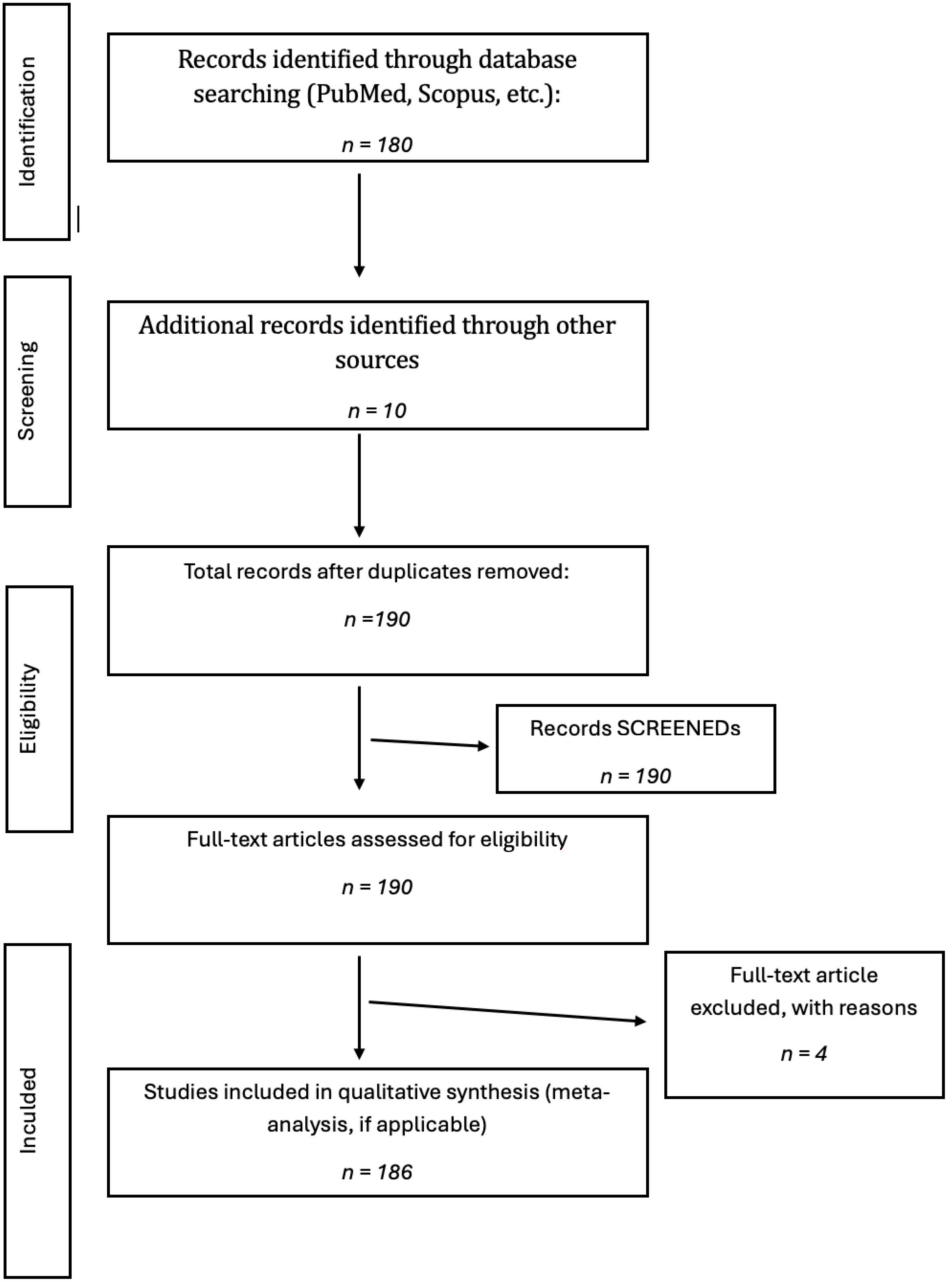
Flow-chart PRISMA.

To ensure a multidisciplinary and translational approach, evidence from diverse study designs was considered, including randomized controlled trials evaluating infant formula composition and microbiota-modulating interventions, observational and cohort studies examining feeding practices and health outcomes, microbiome sequencing studies (e.g., 16S rRNA gene sequencing and metagenomic analyses) identifying microbial patterns associated with different feeding modalities, and policy-oriented reports addressing health inequalities, caregiver support, and environmental determinants of infant nutrition (Figure 2).

## 3 Breast milk, gut microbiota, and infant growth

### 3.1 Human milk: composition, roles, and biological significance

Human milk represents the reference model for nutrition in the first months of life and is recommended as the exclusive nutritional source for up to 6 months. It is a highly dynamic and complex liquid tissue, consisting of 85% water and 15% solids. In addition to macro- and micronutrients, it contains many bioactive components (important bioactive constituents include hormones, growth factors, immune cells, stem cells, microRNAs, extracellular vesicles, microorganisms, human milk oligosaccharides (HMOs), antibodies, and components of the complement system) that perform anti-inflammatory, immunomodulatory, anti-infective, probiotic, and prebiotic functions (10).

Numerous studies have shown that exclusive breastfeeding is associated with a significant reduction in allergies and respiratory infections in the early postnatal period (11). The composition of breast milk evolves through three main phases: colostrum, transitional milk, and mature milk. This progression responds to the infant’s changing nutritional needs. Colostrum, the first form of milk secretion, provides essential elements for initiating growth and immune protection in the newborn. 100 mL contains an average of 50–60 kcal, 14–16 g of protein, 50–62 g of carbohydrates, and 15–20 g of fat. In mothers of preterm infants, the protein content is further increased to support the increased growth demands (12). Approximately 2 weeks after birth, milk reaches the mature stage, characterized by a higher lipid content, a reduction in protein, and a lower presence of immunomodulatory factors. Mature milk contains approximately 6.9–7.2% carbohydrates, 0.8–0.9% protein, 3–5% fat, and 0.2% minerals. It is particularly rich in lactose, water, and B vitamins (especially B1 and B6) (13, 14). Milk composition, however, is subject to individual variability in relation to multiple infantile and maternal factors, as well as breastfeeding methods. Among infantile factors, the most prominent relationships are prematurity, birth weight, sex, birth mode, sibling, and antibiotic exposure. Moreover, among relevant maternal factors are ethnicity, BMI, diet, lifestyle, psychophysical state, and antibiotic exposure. And finally, the direct or indirect breastfeeding method is also relevant (15).

### 3.2 The human milk microbiome

An infant of approximately 4 months consumes 800–850 mL of breast milk daily, containing a total of 10^5^–10^7^ microbial cells. The milk microbiota is highly biodiverse and is the result of a continuous exchange between the mammary gland, the infant’s oral cavity, and the maternal intestinal tract. This bidirectional interaction is supported by three main mechanisms: the enteromammary pathway, retrograde flow, and the oromammary pathway (16).

- Enteromammary circulation describes the physiological passage of maternal intestinal bacteria to the mammary gland, with subsequent transmission to the newborn during breastfeeding (17).
- Retrograde flow results from sucking: a portion of the neonatal oral microbiota reaches the mammary ducts, contributing to microbial diversity (e.g., *Streptococcus*) (15).
- Observations of oral microorganisms in expressed milk suggest an additional oromammary route, potentially involving the transfer of bacteria from the maternal mouth to the breast (18).

Human milk typically contains genera such as *Streptococcus, Staphylococcus, Pseudomonas, Rothia, Bifidobacterium, Veillonella, Bacteroides, Enterococcus*, and many others, up to a total of approximately 590 identified genera (19). Their composition reflects maternal health status; for example, antibiotic use has been associated with a marked reduction in *Bifidobacteria* (20, 21). A BMI > 30 kg/m^2^ is also correlated with lower microbial diversity (22, 23). The method of breastfeeding also influences the composition of the microbiota, as direct breastfeeding is associated with a greater presence of oral microorganisms, such as *Veillonella*, while indirect breastfeeding (expressed milk) shows a greater presence of environmental microorganisms, such as *Pseudomonas* and *Enterococcaceae*, and a reduction in *Bifidobacteria* (24).

### 3.3 Bioactive components of human milk

Breast milk provides a wide range of bioactive molecules with immunomodulatory, endocrine, metabolic, and neurotrophic functions. These include immunoglobulins, complement components, lactoferrin, lysozyme, bacteriocins, antimicrobial peptides, mRNA, HMOs, growth factors, cytokines, and stem cells (25).

Lactoferrin, abundant in colostrum, binds iron and has strong antimicrobial properties. Immunoglobulins such as secretory SIgA and SIgG represent the most abundant forms and protect the intestinal epithelium from pathogen adhesion. IgG, plays a critical role in shaping infant gut immunity. Ingested during the first week of life, maternal IgG binds gut bacteria and modulates microbiota-dependent adaptive immune responses weeks later, after weaning. These antibodies also limit inappropriate neonatal responses to dietary antigens, demonstrating that maternal IgG tunes early mucosal immunity and helps maintain intestinal homeostasis during development (26). Cytokines such as members of the TGF-β family are particularly represented and contribute to the induction of antigen-specific Treg cells, feeding tolerance, and the regulation of inflammation. TGF-β2 in particular promotes epithelial repair (27). Fat globule membranes (MFGMs) such as phosphatidylcholine and sphingomyelin support neurodevelopment, with choline being a precursor to acetylcholine, and sphingomyelin contributing to myelination (28). Moreover, recent studies have demonstrated that complement components in breast milk play a direct role in shaping the neonatal gut microbiota and providing immune protection. In weanling mice, pups fed on complement-deficient milk were highly susceptible to *Citrobacter rodentium* infection, whereas those nursed on complement-containing milk survived. Breast milk complement selectively lyses specific gram-positive commensals through a C1-dependent, antibody-independent mechanism, thereby modulating gut microbial composition and enhancing protection against environmental pathogens (29).

Understanding the functional role of these components in immune maturation and intestinal physiology in the newborn represents an important research perspective.

### 3.4 Human milk oligosaccharides (HMO)

HMOs are one of the main bioactive fractions of human milk. Although not digestible by human enzymes, they are readily fermented by the intestinal microbiota, becoming a key substrate for bacterial growth. They are structurally composed of five monosaccharides (Fuc, Glc, Gal, GlcNAc, and Sia) assembled around a lactose core. Individual variability in their composition depends on maternal genetics (particularly the Se and Le genes encoding FUT2 and FUT3), nutritional status, and environmental factors (30). HMOs resist digestion and reach the colon, where they exert numerous beneficial effects, such as protection from pathogens through glycocalyx mimicry, immune modulation, prebiotic function, and contribution to neurocognitive development (31). Fucosylated HMOs, for example, inhibit the adhesion of pathogens such as *H. pylori, C. jejuni*, and *E. coli* (32) Their prebiotic role is particularly evident in supporting the growth of specific *Bifidobacteria*, including *Bifidobacterium breve*, *B. longum* subsp. *infantis* and *B. bifidum*, not surprisingly defined as infant-type bifidobacteria (33, 34). Fermentation of HMOs leads to the production of SCFAs, particularly butyrate, which is essential for enterocytes and has anti-inflammatory and immunomodulatory properties (35).

### 3.5 Interaction between HMOs, microbiota, and brain

HMOs also influence the maturation of the microbiota–gut–brain axis (MGBA). Sialic acid, highly abundant in milk, is present in brain tissue and participates in neuronal communication. Jorgensen et al. have shown that infant intake of sialylated and fucosylated HMOs is related to better language scores at 18 months, while non-secretory mothers show negative associations (36). In animal models, 2′-FL (2′-fucosyllactose) supplementation improves learning and memory (37). Modulation of the microbiota by HMOs can affect the serotonergic system and the kynurenine-tryptophan pathway. The use of *Bifidobacterium longum* and *Lactobacillus helveticus* reduces the kynurenine/tryptophan ratio and improves depressive symptoms (38). Low levels of *Bifidobacteria* are observed in neurodevelopmental disorders, such as ASD (39). Similarly, patients with Parkinson’s disease and depression have reduced concentrations of SCFAs and butyrate-producing bacteria, such as *Roseburia* and *Faecalibacterium* (40). The bifidogenic effect of HMOs could therefore have potential therapeutic applications.

GABA, produced predominantly by *B. adolescentis*, also plays a central role in the MGBA. A dysbiotic microbiota, depleted of butyrate- and GABA-producing bacteria, may contribute to cognitive deficits (41). HMOs, by promoting the expansion of *Bifidobacteria*, could restore these alterations, opening prospects for the treatment of neurodegenerative and neurodevelopmental diseases (42).

The activities of bioactive components of human milk, particularly HMO, are largely based on their ability to modulate the neonatal gut microbiota, promoting eubiosis, microbial cross-feeding, and the production of key metabolites, including SCFAs and neurotransmitters. These effects are not limited to the gastrointestinal tract but also involve the central nervous system, suggesting potential therapeutic applications in neurodegenerative and neurodevelopmental disorders.

## 4 Impact of formula feeding on infant health: short- and long-term consequences

It is estimated that expanding breastfeeding to a near-universal level could prevent 823,000 deaths per year in children under 5 years of age and 20,000 deaths per year from breast cancer in mothers (2). Several studies have demonstrated the beneficial effects of breastfeeding in resolving the lack of maternal microbial colonization at birth due to cesarean section (43, 44) and in mitigating the severe delay in microbial colonization that characterizes preterm birth (45). Microbial colonization in formula-fed infants compared to breastfed infants is characterized by lower microbial diversity and lower abundance of *Bifidobacterium* and *Lactobacillus*, a higher presence of potentially harmful bacteria such as *Enterobacteriaceae, Bacteroidaceae*, and *Clostridiaceae*, and metabolic profiles associated with greater intestinal inflammation (46).

### 4.1 Short-term consequences

The absence of maternal antibodies, complement components, HMOs, and immune cells in standard infant formulas reduces the infant’s ability to fight common pathogens (31). Therefore, infants fed formula show a higher incidence of gastrointestinal, respiratory, and ear infections than breastfed infants (47, 48).

Furthermore, formula, although formulated to meet energy needs, can lead to faster growth rates and changes in body composition, with greater accumulation of fat mass (49).

### 4.2 Long-term consequences

Infants fed exclusively formula, due to lack of exposure to HMOs and bioactive compounds, show reduced epigenetic modulation of metabolism and the gut-brain axis and become more susceptible to so-called non-communicable diseases such as obesity, type 2 diabetes, hypertension, and metabolic syndrome in adulthood (50). The absence of immunomodulatory factors and natural prebiotics in standard formulas compromises the formation of early immune tolerance, leading to atopic disease, eczema, asthma, and food allergies (51). Finally, numerous studies suggest differences in cognitive tests between breastfed and formula-fed children. All of this can be attributed to the docosahexaenoic acid (DHA), choline, and bioactive lipids present in breast milk, which support myelination and the development of executive functions (52–54), although some of these differences may derive from socioeconomic and environmental factors.

Considering this evidence, it is clear that the absence of breast milk represents not only a nutritional variation but a profound change in the child’s ecological, immunological, and microbiome trajectories.

## 5 When breastfeeding is not possible: strategies to bridge the gap

### 5.1 Microbiota health does not depend on milk alone

The newborn’s microbiota is formed through many pathways: contact, nutrition, environment, and emotional relationships. The maternal role remains central. Metagenomic studies show that, already in the first hours of life, the newborn’s microbial composition significantly reflects the maternal intestinal, vaginal, cutaneous, or environmental microbiota, depending on the delivery method and regardless of nutrition (55–57).

The postnatal phase is characterized by high microbial plasticity: the microbiota in the first few months shows low stability and continuous evolution that responds to physiological intestinal maturation rather than the type of milk consumed. Longitudinal studies have shown that, in the first 6 months of life, bacterial diversity and composition follow common trajectories between breastfed and non-breastfed infants, with differences that tend to diminish over time (58, 59). At the same time, the development of the intestinal barrier and mucosal immune system, with the progressive expression of IgA, mucins, and pattern-recognition receptors, represents a central driver of microbial maturation and proceeds according to an intrinsic biological program, independent of breastfeeding (60, 61).

**Figure 3:**
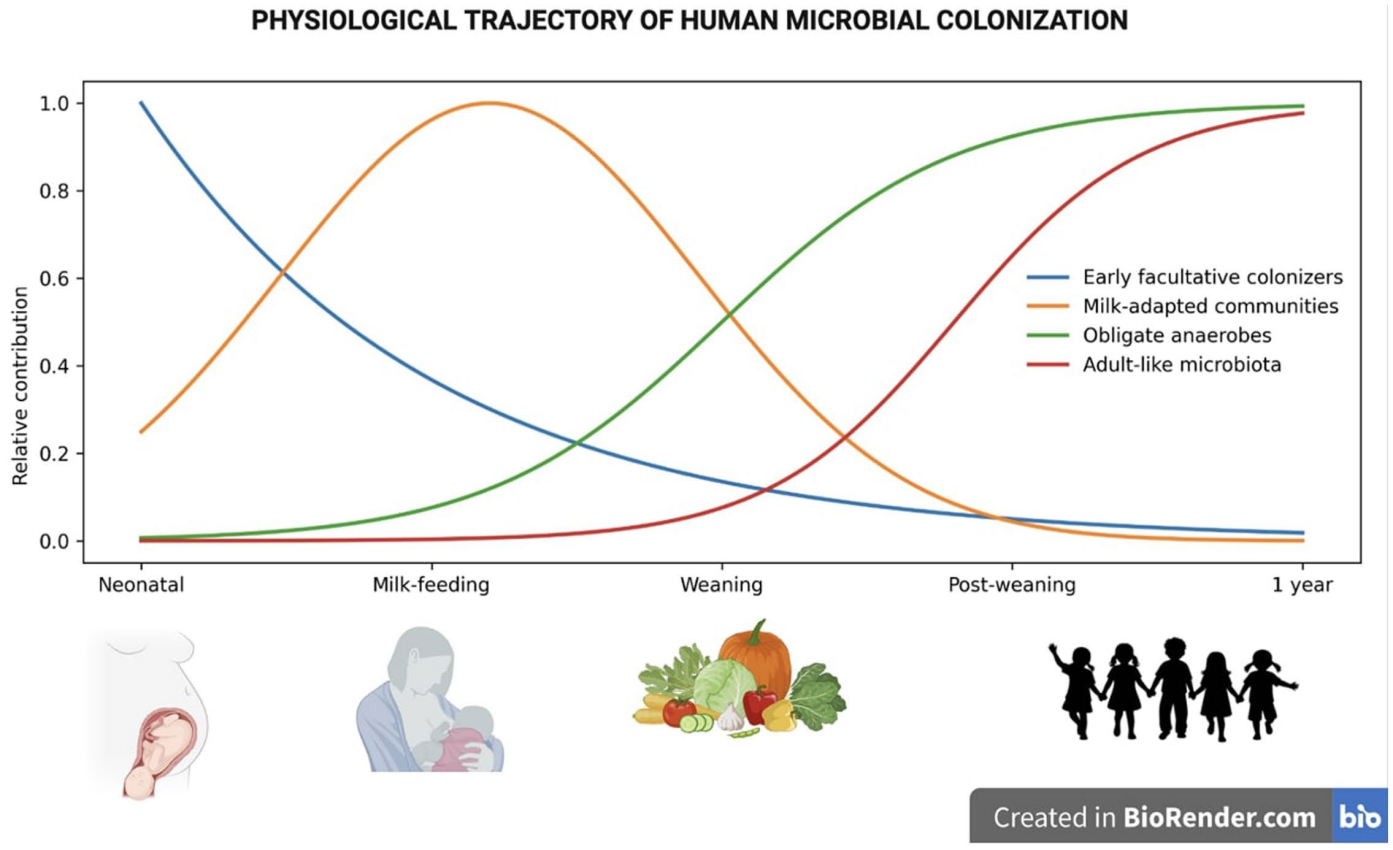
Conceptual ecological trajectories of infant gut microbiota development. Curves represent schematic, non-quantitative trajectories describing major ecological configurations of the gut microbiota across early-life developmental stages.

This evidence confirms that the construction of the infant microbiota results from a multifactorial, biologically redundant process, not tied to a single element. For this reason, when breastfeeding is not possible, “all is not lost,” but microbial colonization can still proceed through natural physiological pathways that remain active and highly modulable (Figure 3).

### 5.2 Targeting infant health with bioactive-enriched formulas

Modern infant formulas, available in liquid, powdered, or ready-to-use forms, are predominantly derived from cow’s milk, which has a higher protein and lipid content than human milk. This difference has been associated with a more rapid increase in adiposity in the first months of life and a greater risk of obesity in later life (62). To make formulas more like the functional profile of breast milk, the industry has progressively introduced bioactive components capable of favorably modulating the neonatal microbiota (which we will discuss in the following chapters); however, beyond nutritional aspects, the quality of the formula packaging and preparation methods is also critical. Exposure to compounds such as bisphenol A (BPA) and phthalates, frequently released from plastic materials, has been associated with potential adverse effects on the endocrine system and immune development (63–65). For this reason, it is advisable to use formulas packaged in materials free of these substances and to use glass or stainless-steel bottles (66).

Overall, choosing formulas supplemented with pre-, pro-, and postbiotics, combined with safe preparation practices, helps promote more physiological microbial and immune development, bringing the impact of formula feeding closer to that of breastfeeding.

### 5.3 Rationale choice of prebiotics, probiotics, and postbiotic in infant formulas

The term “prebiotic,” according to the definition of the International Scientific Association for Probiotics and Prebiotics (ISAPP), refers to a substrate selectively utilized by host microorganisms that can provide clinically relevant benefits (67). Interest in prebiotics has grown significantly during the neonatal period, as they represent one of the primary nutritional tools for guiding the maturation of the intestinal microbiota in the early stages of life. Selectivity is a fundamental criterion: although all prebiotics are fibers, not all fibers possess prebiotic properties. Compounds such as cellulose and pectins, although fermentable, do not selectively target specific microbial taxa and are therefore not classified as such. In newborns, the most studied and used prebiotics are galactooligosaccharides (GOS) and fructooligosaccharides (FOS), frequently added to infant formula to mimic the physiology of breast milk (68). GOS, available in *α* and *β* forms (the latter synthetic and the most widely used in pediatrics), selectively promotes the growth of *Bifidobacterium* and *Lactobacillus*, to the detriment of potentially pathogenic species such as *Clostridium* spp. Evidence suggests that their supplementation in the first weeks of life promotes bifidobacterial colonization, helps reduce the incidence of constipation, and supports immune maturation through the modulation of cytokines and SCFA production (69). An *in vitro* study conducted by Marzorati et al. (70) examined the prebiotic activity of GOS. Exposure of samples to the supplement resulted in a marked enrichment of microbial populations considered beneficial, accompanied by an increase in the production of short-chain fatty acids and lactic acid. At the same time, a decrease in branched fermentative compounds, a reduction in pH, and a drop in ammonium concentrations were observed, suggesting a metabolic profile more favorable to intestinal health (70). Moreover, an experimental work conducted by Akbari and colleagues highlighted that GOS exerts a protective role on the integrity of the intestinal barrier in a Caco-2 cell model exposure to deoxynivalenol (mycotoxin) compromised the continuity of the epithelial monolayer, while treatment with GOS counteracted this effect by promoting a more rapid restoration of tight junctions (71). Furthermore, GOS supplementation also reduced the release of CXCL8, an important pro-inflammatory mediator, suggesting a potential contribution to the modulation of enteric inflammatory responses (72).

FOS, naturally present in some plant sources, are similarly used in infant formulas due to their ability to increase the proportion of *Bifidobacteria* and intestinal lumen acidification. In pediatrics, FOS supplementation has been associated with improved intestinal function, increased bowel frequency, and reduced need for therapies for functional constipation. A study conducted by Grüber et al. (73) analyzed the effects of a formula enriched with a prebiotic combination characterized by a 9:1 ratio of GOS to long-chain FOS, also supplemented with 1.2 g/L of acidic oligosaccharides derived from pectin. The overall concentration of prebiotics in the formula was 6.8 g/L. The results showed that the intake of this formulation in the first year of life was associated with a 44% reduction in the incidence of atopic dermatitis in infants not considered at high allergic risk. However, this benefit was not maintained over time; once prebiotic supplementation was suspended, the protective effect against eczema was no longer detectable in preschool age, suggesting that the immunomodulatory impact of the formula requires continuous exposure to be sustained (73).

Some subclasses, such as xylooligosaccharides (XOS), are selectively fermented by *Bifidobacterium adolescentis*, while isomaltooligosaccharides (IMO) favor beneficial taxa such as *Lactobacilli* and *Bifidobacteria* (74).

Overall, prebiotics represent a promising tool for modulating the neonatal microbiota in a eubiotic sense, supporting intestinal maturation, the mucosal barrier, and the immune response. The use of GOS, FOS, and other selective fermentable compounds in neonatal formulas has proven safe and effective; however, expanding the use of new classes of prebiotics requires further multicenter, longitudinal clinical studies to define their efficacy and safety profile in the first months of life.

However, multivariate HMO mixtures represent the most physiologically relevant prebiotic candidates for infant formulas, as they better approximate the ecological functions of human milk in shaping bifidobacteria-dominated microbial communities (75, 76). Therefore, formula feeding results in profoundly different infant microbiota configurations, with a marked reduction in infant-type *Bifidobacteria* and a greater expansion of opportunistic taxa such as *Enterobacteriaceae, Clostridiaceae*, and *Streptococcaceae*, with potential implications, especially for immunity, inflammation, and susceptibility to infections (77). The addition of probiotics to formula, or their external supplementation, therefore, represents an attempt to mitigate this biological gap by modulating the microbiota in a “pro-bifidogenic” manner. However, to do so rationally, it is necessary to understand how the different strains behave in an environment devoid of HMOs and rich in GOS and FOS, as in standard infant formula. Not all *Bifidobacteria*, in fact, possess the same metabolic versatility (78).

Among the infant-type *Bifidobacteria*, *B. infantis* is certainly the most efficient user of HMOs, possessing complex systems for importing and metabolizing these polysaccharides intracellularly. This strain, therefore, has a great capacity to colonize the intestine of an infant fed with human milk, but has little chance of colonization when fed with standard formulas that are free of HMO (79). Consequently, its introduction, or supplementation in standard milk formulas, which contain GOS and FOS but no HMO, appears biologically unjustified. In an HMO-free environment, *B. infantis* is outcompeted by other *Bifidobacteria* that are better able to metabolize GOS, FOS, and mucin. *B. bifidum* possesses a broad repertoire of enzymes (sialidase, fucosidase, N-acetyl-β-glucosaminidase, and mucinolytic enzymes) that are exocytosed and are capable of degrading both HMO and intestinal mucin, releasing simple sugars that can also be used by other *Bifidobacteria* such as *B. breve* (24). These capabilities also make it suitable for supplementation in infants fed standard formulas, where it can use mucin, GOS, and FOS as its main substrates, contributing to the creation of a more physiological ecosystem. Furthermore, its sialidases, by releasing sialic acid from sialylated oligosaccharides but also from mucin, may be beneficial for brain development (80). *B. breve*, despite being an infant-type strain, shows a limited ability to utilize GOS and FOS and a poor efficiency in mucin metabolization. Consequently, it is only effective when combined with *B. bifidum*, thanks to cross-feeding phenomena (81, 82); when combined, the two strains show strong synergy. *Lactobacilli*, such as *L. reuteri* and *L. rhamnosus*, play a complementary role: they support barrier function, modulate mucosal immunity, produce lactic acid and bacteriocins, and compete with potential pathogens (83). Although they are unable to reconstruct an infant-type community, they can improve mucosal resilience and reduce gastrointestinal disorders (83).

Postbiotics are defined as preparations containing inanimate microorganisms and/or their components that confer a health benefit to the host. They can also be described as deliberately inactivated microbial cells, with or without associated metabolites or cellular components, that contribute to documented health effects. However, the INNOVA 2020 study (84), infants randomized to receive an intervention formula (*n* = 70), containing a heat-inactivated postbiotic (*Bifidobacterium animalis* subsp. *lactis* CECT 8145 BPL1™), a lower total protein content, a reduced casein-to-whey protein ratio, and a doubled docosahexaenoic acid/arachidonic acid ratio compared with a standard formula, experienced a lower incidence of atopic dermatitis as well as fewer episodes of bronchitis and bronchiolitis than infants fed the standard formula (*n* = 70) (*p* = 0.03). Notably, these outcomes were comparable to those observed in breastfed infants (*n* = 70) (*p* = 1.0).

Additional evidence comes from studies on *Bifidobacterium breve* C50 and *Streptococcus thermophilus* ST065, lactic acid–producing bacteria shown to exert anti-inflammatory effects on intestinal cells *in vitro* (85). An infant formula fermented with these two strains, without viable bacteria in the final product, was evaluated in healthy infants (*n* = 464) and compared with a non-supplemented formula group (*n* = 449). Both formulas were administered for 5 months, starting at 4 months of age. Although the incidence of acute diarrhea did not differ between groups, the severity of acute gastroenteritis was reduced in the fermented formula group, as indicated by fewer hospital admissions, lower rates of acute dehydration, reduced medical consultations, and fewer prescriptions of oral rehydration solutions.

A recent systematic review published in *Nature* (86) and registered in the PROSPERO database (CRD42022352405) evaluated the effects of infant formulas supplemented with postbiotics compared with standard formulas. The review included nine randomized controlled trials comprising a total of 2,065 participants and assessed outcomes related to safety, gastrointestinal tolerance, fecal SIgA concentrations, and growth parameters. The authors reported that postbiotic supplementation was associated with a significant increase in fecal SIgA levels, although the certainty of the evidence was rated as very low. No significant differences were observed between groups with respect to the incidence of serious adverse events, gastrointestinal symptoms, including infantile colic, flatulence, diarrhea, vomiting, abdominal pain, and other gastrointestinal disorders, or growth outcomes such as weight gain, length gain, and head circumference growth. Overall, the findings suggest that the addition of postbiotics to infant formula is safe and may confer immunological benefits without adversely affecting growth or gastrointestinal tolerance.

### 5.4 Bioactive formulas or external supplementation? Current technical limitations of supplementation

Probiotic-enriched formulas represent a strategy with advantages related to standardization and safety control (87); however, integrating live probiotics into formulas is difficult. A series of technical challenges exist in industrial production: milk formulas undergo spray-drying, mixing, and homogenization processes that generate thermal, oxidative, and mechanical stress that compromise the viability of microorganisms (88). Even during storage, a progressive loss of viable cells occurs. Furthermore, even after ingestion, probiotics must contend with the problem of surviving gastric acidity and the enzymatic hydrolysis of bile and pancreatic enzymes, making colonization unpredictable. These limitations are pushing for new technological solutions (microencapsulation, nanoencapsulation, hybrid formulations with combined use of prebiotics, probiotics, and postbiotics) (89, 90). Studies conducted to date show great variability in clinical effects: these formulas tend to increase the abundance of *Lactobacilli* and some *Bifidobacteria*, but fail to reconstruct the structure dominated by infant-type *Bifidobacteria* typical of breastfed infants (91). Furthermore, the impact on colic, infections, and immune modulation remains modest and uneven (92). External supplementation allows for a more targeted and personalized choice of metabolically more suitable strains or functional consortia. However, the quality of available products is not always uniform, viability is not always guaranteed, and colonization can be inconsistent.

Postbiotics offer a solution to many of these critical issues. These are preparations of inactivated microorganisms and/or their bioactive metabolites, which maintain immunomodulatory, anti-inflammatory, and mucosal maturation-supporting properties, without requiring viability or colonization (93). Because they are not viable organisms, they are stable to heat and storage. Studies indicate a good safety profile, especially in more fragile infants, and potential benefits in regulating barrier and endothelial function, metabolism, and immunomodulation, as well as sIgA production and mucosal reactivity (86, 94).

Postbiotics, therefore, represent one of the most promising areas of development for future formulas.

### 5.5 Balancing hygiene: avoiding over-sterilization

In 2002, Ownby et al.’s prospective cohort study of children in Detroit, Michigan, reported that exposure to cats and dogs in the first year of life significantly reduced the risk of allergic sensitization between the ages of 6 and 7 years (95). Since then, numerous studies have supported the hypothesis that childhood exposure to excessive sanitization and hygiene regimens is associated with an increased risk of, and the onset of, childhood immune system diseases (96). To effectively support the establishment of a healthy gut microbiota in newborns, a balanced approach to home hygiene is strongly recommended: this involves regular cleaning without excessive disinfection, maintaining rigorous personal hygiene (especially handwashing), and promoting safe and natural exposure to environmental microbes. Research consistently suggests that the use of harsh disinfectants and detergents should be limited, as they are associated with reduced infant gut microbiota diversity and increased colonization by potentially harmful bacteria, such as *Clostridioides difficile* (97). Furthermore, natural microbial exposure is recommended, as the presence of siblings, pets, and contact with natural environments (where safe) have been associated with greater microbiota biodiversity and proven immunomodulatory benefits. This contrasts with an excessively sterile environment, which can limit exposure to beneficial microbes (98, 99).

### 5.6 Skin to skin and emotional contact in the mother-infant dyad

Early physical contact is a fundamental determinant of newborn health, with effects that, beyond the relational sphere, involve neuroendocrine, immune, and physiological dimensions closely linked to the maturation of the microbiota. A Cochrane review (100) on the topic highlighted that immediate and prolonged skin-to-skin contact improves cardiorespiratory stability, promotes more effective thermoregulation, reduces crying, and supports the newborn’s behavioral regulation. A pilot study conducted in 2023 (101) demonstrated that daily skin-to-skin contact with parents in preterm infants is associated with significant changes in heart rate variability (an indicator of autonomic nervous system maturation and enhanced vagal tone), suggesting beneficial physiological effects related to autonomic modulation during the early days of life. Because vagal tone, systemic inflammation, and immune maturation are closely linked to the development of the gut microbiota, these results suggest that skin-to-skin contact represents a non-nutritional intervention capable of indirectly influencing microbial trajectory. In parallel, metagenomic studies have shown that physical contact between mothers and infants allows the transfer of cutaneous and oral bacterial communities, contributing to the colonization of commensal species considered founders of the neonatal microbiota and reducing the likelihood of colonization by opportunistic environmental microorganisms (102). This is particularly relevant for mothers who cannot breastfeed: even in the absence of breast milk, physical closeness and babywearing are effective tools to support the child’s microbial and immunological health.

### 5.7 The importance of supporting the maturation of the oral microbiota

The oral microbiome is a complex community, and its composition and persistence are closely influenced by diet, oral hygiene practices, and salivary flow (103, 104). Several studies investigating the composition of the oral microbiome using advanced DNA sequencing techniques have found that the major phyla present in the oral cavity are: *Firmicutes, Bacteroides, Proteobacteria, Actinobacteria, Fusobacteria*, and *Spirochaetes* (105–108).

Although the origin of the neonatal oral microbiome is poorly understood, it is clear that the richness and changes in the composition of the neonatal oral microbiome impact short- and long-term health (109, 110). It is assumed that the initial development and maturation of the neonatal oral microbiome are largely determined by microbiome exchanges between mother and offspring during and after birth (111, 112). Growing evidence demonstrates that the neonatal oral microbiome has a prenatal origin (113, 114). There is evidence that the newborn’s oral microbiome is influenced primarily by the mode of delivery (115, 116), and several studies have demonstrated its impact on the newborn’s future health. Indeed, the influence of cesarean section on the composition of the microbiome and its association with an increased incidence of celiac disease, asthma, type 1 diabetes, and obesity has been demonstrated. The use of antibiotics during pregnancy can also increase the risk of childhood diseases in the offspring (20, 117).

The newborn’s oral microbiota is initially very sparse but rapidly populates with bacteria, primarily through vertical transmission from the mother (vaginal, oral), but also through birth (cesarean vs. natural) and environmental factors. This development leads to the development of diverse communities (such as *Streptococcus, Neisseria*) that influence future health and susceptibility to diseases such as tooth decay or infections, with diversity increasing and stabilizing over time (118).

The species characterized by high pathogenicity are: *Aggregatibacter actinomycetemcomitans, Porphyromonas gingivalis, Tanarella forsythia*, and *Treponema denticle*. These are joined by species with lower pathogenicity, such as *Prevotella intermedia* and *Fusobacterium nucleatum*.

Over the past 20 years, scientific literature has shown growing interest in demonstrating that periodontal disease and its bacteria can be responsible for pregnancy complications. It is known that a woman’s oral health and oral microbiome can directly influence her pregnancy and her developing fetus: if the mother is affected by periodontal disease, she has a higher risk of giving birth prematurely, giving birth to a low birth weight, preeclampsia, and a 3.4 times greater risk of giving birth prematurely plus giving birth to a low birth weight (119). Furthermore, some authors have shown that in the case of oral diseases (gingivitis or periodontitis), bacteria present in the oral cavity can reach the amniotic fluid through a transient bacteremia, which indicates that maternal microbes can be transmitted to the amniotic fluid through the blood (120, 121). Thus, although there is still not enough evidence to conclude, it has been proposed that periodontal pathogens or their products somehow reach the placenta and spread further to the fetus (122). Two pathogenic mechanisms have been proposed to explain the connection between maternal oral dysbiosis and placental and fetal biology: the direct pathway is based on the presence of gram-negative anaerobic bacteremia originating in the gingival biofilm, while the indirect pathway involves the production of pro-inflammatory markers entering the bloodstream from the gingival submucosa (123, 124). The maternal oral microbiota represents a major source of microbial inoculum for the newborn in the early stages of life, significantly contributing to the colonization of the oral cavity and, due to anatomical and functional continuity, the upper respiratory tract. Already at birth, and particularly during vaginal delivery, the newborn is exposed to a complex maternal microbial community, which includes not only the vaginal and intestinal microbiota, but also the oral microbiota, transferred through direct contact, secretions, and early caregiving practices (125). The maternal oral microbiota is dominated by genera such as *Streptococcus, Veillonella, Neisseria, Haemophilus*, and *Actinomyces*, many of which are among the first colonizers of the neonatal oral cavity and play a key role in structuring the initial microbial communities (126).

Molecular typing studies have demonstrated significant phylogenetic similarity between maternal and neonatal oral strains, suggesting direct vertical transmission, occurring not only at birth but also in the postnatal period through skin-to-skin contact, saliva, kissing, and breastfeeding (127).

The quality of the maternal oral microbiota appears to be particularly important. Conditions such as periodontitis, active caries, or oral dysbiosis have been associated with an altered composition of the infant’s oral microbiota, with a greater abundance of pathobionts and a reduced presence of bacteria. Beneficial commensals, especially non-pathogenic oral streptococci (128). These alterations could influence not only future oral health but also upper airway colonization and the maturation of respiratory mucosal immunity. The maternal oral microbiota also contributes to the early immune education of the newborn. Vertically transmitted oral bacteria participate in the induction of immunological tolerance and the regulation of Th1/Th2/Th17 responses at the mucosal level, promoting an immune balance that could have long-term effects on the risk of recurrent respiratory infections and inflammatory diseases (129, 130). In this context, the oral microbiota represents a potential link between the immune system, oral-pulmonary system and early immunological programming. Overall, the maternal oral microbiota emerges as a significant biological determinant in the development of the neonatal oral-respiratory microbiota. Its composition and stability could represent an early preventative target, paving the way for strategies to promote maternal oral health as an indirect modulatory intervention. The oral microbiota in early pregnancy is enriched with several pathogens (*Porphyromonas gingivalis and Aggregatibacter actinomycetemcomitans, Candida*), likely due to increased levels of progesterone and estrogen. These data further support the importance of preventive dentistry during pregnancy for both the mother and the fetus. It is therefore important to know the factors that can modify the maternal microbiota and, consequently, influence the microbial colonization of the fetus (Table 1). However, to maintain a healthy microbiota, the literature highlights essential strategies such as brushing and flossing at least twice a day, choosing gentle toothpastes and mouthwashes and avoiding overuse, following a diet rich in fiber and probiotics (vegetables, fruit, unsweetened yogurt, kefir) and low in sugar, using oral probiotic supplements, if recommended, monitoring blood glucose levels, especially in diabetics, avoiding smoking and managing stress, and having regular dental checkups (131, 132). Consequences of dysbiosis include oral diseases (caries, gingivitis, periodontitis, canker sores) and systemic diseases (potential links to heart, lung, and even cancer) (133).

**Table 1:** Environmental factors that can modify the maternal microbiota and influence fetal microbial colonization.

Environmental factor	Description
Periconceptional maternal diet	The maternal diet during the periconceptional period (from 1 month before to 3 months after conception) plays a key role. The growth of *Bacteroides* (essential for the development of the infant microbiota) is promoted by the intake of foods rich in non-digestible fibers such as vegetables and cereals. In contrast, *Firmicutes*, found in higher-than-normal levels in the microbiota of obese mothers, infants born by cesarean section, or overweight children aged 1–3 years—proliferate in the presence of fats and simple sugars (124).
Weight gain and body mass index during pregnancy	Maternal weight gain and body mass index (BMI) during pregnancy can influence the composition of the maternal microbiota and, consequently, fetal microbial colonization (55).
Medications taken during pregnancy	Approximately 80% of drugs prescribed during pregnancy are antibiotics. These cause alterations in the microbiota across multiple ecosystems, potentially affecting the development of the fetal and neonatal microbiota and predisposing the child to obesity, metabolic syndrome, or autoimmune manifestations (from atopic dermatitis to allergic asthma) (184).
Type of delivery and feeding	The mode of delivery (vaginal birth or cesarean section) and the type of infant feeding (breastfeeding or formula feeding) significantly influence early microbial colonization (117).
Intrauterine microbial development	The first steps in the development of the oral ecosystem already occur during intrauterine life. This ecosystem is important not only for local health but also for the health of interconnected organs and systems (116).

## 6 Complementary feeding as a microbial and metabolic transition window

### 6.1 Impact of complementary feeding

At birth, the newborn has an immature gastrointestinal and immune system. With the introduction of complementary feeding around 6 months of age and the gradual continuation of breastfeeding until 2 years, the gut microbiota undergoes a major ecological transition. The early predominance of *Actinobacteria* and *Proteobacteria* decreases, while adult-like microbial configurations emerge, characterized by an expansion of *Firmicutes* and *Bacteroidetes*, including key taxa such as members of the *Ruminococcaceae* family, *Bacteroides* spp., and *Akkermansia muciniphila*, together with a progressive increase in microbial biodiversity (134). This shift reflects the greater availability of complex carbohydrates and fermentable fibers (such as resistant starches, FOS, GOS, and inulin), which favor SCFA-producing bacteria, contributing to eubiosis and mucosal integrity. Protein-rich foods, on the other hand, promote proteolytic metabolism, including *Clostridium* and *Streptococcus* (135) (Figure 3).

Similarly, dietary diversity in early childhood influences not only microbial composition but also the development of taste preferences (136). Fetal and neonatal exposure to aromatic compounds present in amniotic fluid and breast milk contributes to shaping food choices during weaning (137). Evidence also highlights how the quality of nutrition in the first 1,000 days impacts long-term metabolic risk: for example, exposure to reduced sugar consumption in early life is associated with a lower incidence of cardiometabolic diseases in adulthood (138). Emphasis should be placed on providing fresh, minimally processed foods from controlled supply chains, favoring seasonal and whole-grain products. Diets rich in dietary fiber and phytonutrients support the growth of beneficial bacterial communities, particularly fermentative taxa producing SCFAs, which are crucial for intestinal barrier integrity and systemic inflammation modulation (139). Moreover, careful consideration of cooking and storage methods is essential to preserve nutritional value and limit exposure to harmful compounds. Gentle cooking techniques such as steaming, controlled boiling, or baking reduce the loss of thermolabile micronutrients and prevent the formation of potentially harmful molecules like advanced glycation end products (AGEs) (140). Safe cookware and storage materials, including stainless steel, borosilicate glass, and cast iron, should be prioritized, while avoiding plastics, aluminum, and non-certified non-stick surfaces to minimize exposure to endocrine-disrupting chemicals such as phthalates, bisphenols, and PFAS (141).

Childhood is also a critical period for the risk of malnutrition, due to factors such as food insecurity, poverty, inadequate complementary feeding, and low birth weight. Globally, childhood malnutrition takes on three facets: undernutrition, micronutrient deficiencies, and overnutrition, with millions of children suffering from stunted growth, wasting, or excess weight. The most recent joint estimates from UNICEF, WHO, and the World Bank indicate that, in 2020, childhood malnutrition remained a major global problem: nearly 150 million children under five were stunted, over 45 million were wasted, and approximately 39 million were overweight. Data from a survey conducted in Rwanda also show a particularly marked increase in rickets, ranging from just over 10% in children under 6 months to nearly 50% in the 18–23-month age group (142). This marked variation highlights the urgent need for specific nutritional strategies across the different stages of early childhood, with comprehensive interventions. Micronutrient deficiencies, often present even in contexts of high energy availability, are particularly insidious and frequently associated with adverse outcomes in children with obesity, where they contribute to the development of metabolic complications (143).

However, in preterm infants and those with extremely low birth weight (ELBW), appropriate early nutritional management is particularly critical. These infants represent a vulnerable population with specific nutritional requirements that differ from those of term neonates. Early initiation of complementary feeding in preterm infants has been associated with an increased risk of accelerated weight gain, as well as a higher incidence of allergy and anemia (144). Conversely, postponing weaning beyond 7–10 months of postnatal age may be linked to a greater likelihood of food refusal and avoidant feeding behaviors (145). With regard to the introduction of solid foods in preterm infants, two key considerations should be addressed. First, when acceptance and intake of semi-solid foods remain insufficient, particular attention should be given to micronutrient provision. In this context, iron and multivitamin supplementation may be beneficial to ensure adequate micronutrient intake (146). Second, if catch-up growth has not been achieved at the time of weaning, strategies to promote a higher protein and energy intake should be implemented, including the use of appropriate formulas or targeted complementary foods (146).

Overall, the perinatal phase and the first year of life constitute a critical window in which microbial colonization, habits, and nutritional habits and the metabolic bases that will influence the child’s health in later years (147).

### 6.2 Home-prepared versus commercial foods

**Figure 4:**
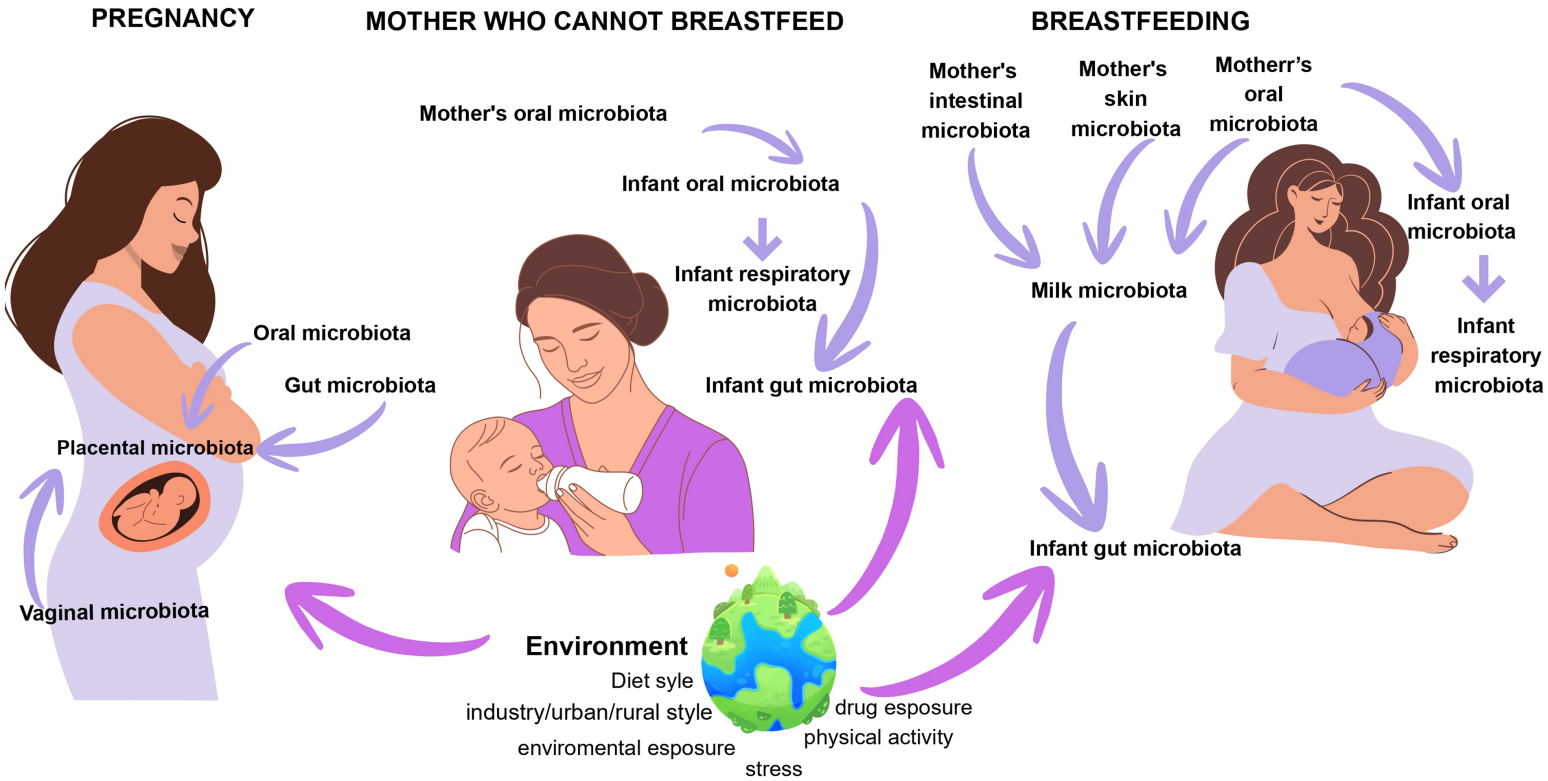
The image illustrates how, during pregnancy, breastfeeding, and for mothers who cannot breastfeed, the maternal microbiota is transferred to the newborn. In each condition, epigenetics mediates the impact of microbial signals and environmental factors on the newborn’s microbial expression, influencing the development of the immune, neural, and metabolic systems.

Home-prepared complementary foods typically offer greater texture variability, higher fiber content, and fewer additives compared to commercially produced products, which are often homogenized and heat-treated, reducing sensory complexity and altering bioactive compounds. Exposure to diverse textures supports the development of oral skills such as chewing, manipulation, and swallowing. Studies indicate that the timely introduction of fruits and vegetables, even in the presence of initial hesitation, enhances acceptance and consumption later in life (148). Delayed introduction of lumpy textures, especially after 9 months, is associated with persistent rejection of structured foods and increased feeding difficulties (149). Healthcare professionals play a pivotal role in guiding caregivers through this transition, fostering balanced diets, oral motor skill development, and long-term health trajectories beyond early childhood (Figure 4).

## 7 Early nutrition, immune system development, and allergy incidence

Susceptibility to allergic and inflammatory diseases cannot be explained solely by genetic determinants. Despite significant advances in understanding the genetic basis of asthma and allergic diseases, the contribution of genetics remains limited, reflecting the multifactorial complexity of these conditions (150). This evidence has guided research toward analyzing early environmental factors as key elements in immune programming. Studies of migrant populations, epigenetic investigations, and microbiota analyses have consistently demonstrated that the environment can profoundly modulate human immune function, influencing the risk of developing immune-mediated diseases throughout life (151, 152). Incomplete or impaired acquisition of immune tolerance is a common pathogenetic mechanism in numerous conditions, including allergies and asthma, autoimmune diseases, chronic infections, and cancers (153). In this context, progressive urbanization and exposure to environmental environments characterized by high levels of chemical pollutants, reduced availability of green spaces, and depletion of plant, animal, and microbial biodiversity are associated with a greater prevalence of immune dysfunction. Reduced contact with natural environments and the environmental microbiota has been linked to the increase in numerous “diseases of civilization,” including allergic diseases and type 1 diabetes (154, 155).

According to the biodiversity hypothesis, exposure to a wide range of microorganisms derived from soil, plants, animals, and natural environments promotes the development of regulatory immune responses and greater immunological tolerance. The loss of biodiversity in natural ecosystems is likely accompanied by a reduction in environmental microbial diversity and a decrease in interactions between the environmental and human microbiota, with potential negative consequences for immune maturation. This phenomenon is particularly relevant in low- and middle-income countries, where the increase in the incidence of chronic inflammatory diseases parallels urbanization and the adoption of Western-style lifestyles (156–158).

In childhood, and particularly in the first years of life, environmental exposure plays a crucial role in shaping the microbiota and immunometabolic programming. The opportunity to interact daily with biodiverse outdoor environments through free play, physical activity, and sensory exploration promotes physiological contact with environmental microorganisms, sunlight, and a variety of evolutionarily relevant ecological stimuli. Experimental and interventional studies have demonstrated that such exposures are associated with an increase in the diversity of the skin and gut microbiota, with an enrichment of beneficial commensal taxa and an increase in the production of immunomodulatory metabolites, such as SCFAs (159–161).

Within this paradigm, living with pets also represents a significant source of microbial exposure. Longitudinal evidence indicates that children exposed early to dogs or cats have a greater diversity of gut microbiota, a more frequent colonization of butyrate-producing bacteria, and an immune profile associated with a lower Th2 polarization, with a consequent reduction in the risk of allergies and asthma in childhood (162). The effect mediated by pets appears largely independent of the type of diet in the first year of life, suggesting a direct role of environmental microbial enrichment in modulating immune trajectories (163, 164). Overall, exposure to nature, understood as interaction with ecosystems rich in microbial biodiversity and controlled coexistence with domestic animals, emerges as a fundamental environmental determinant for the maturation of the microbiota and the immune system at an early age (165). In particular, in children who cannot benefit from breastfeeding, such exposures can represent an important complementary lever to promote correct immunometabolic development and reduce the risk of long-term immune dysfunction.

## 8 Effects of antibiotics on the microbiota

Antibiotic therapy has been pivotal in reducing mortality from bacterial infections, but it profoundly impacts the intestinal microbiota, affecting both short- and long-term health. Antibiotics reduce microbial diversity, compromise ecosystem resilience, and favor the proliferation of opportunistic pathogens. Studies, including Dethlefsen et al. (166), show that bacterial diversity can remain altered for months after treatment. Different microbial groups are affected in distinct ways. *Bacteroidetes* are often drastically reduced, recovering slowly and incompletely, while *Proteobacteria* frequently increase due to the competitive advantage created by the loss of commensal species. *Actinobacteria*, particularly *Bifidobacterium,* are highly sensitive to macrolides and tetracyclines. Within the *Firmicutes*, some families, such as *Lachnospiraceae*, are lost, whereas others, including *Enterococcus*, tend to proliferate. The depletion of key butyrate-producing genera, such as *Faecalibacterium, Roseburia*, and *Agathobacter*, compromises the intestinal barrier and promotes a pro-inflammatory state (167, 168).

Antibiotics also profoundly alter microbiota function. They reduce the production of SCFAs, especially butyrate, which is essential for intestinal epithelial health, increase intestinal pH, and make the gut more susceptible to opportunistic infections, such as *Clostridioides difficile* (97). They can modify carbohydrate, lipid, and drug metabolism and reduce the abundance of genes involved in vitamin biosynthesis, including vitamins K, B12, and folate. Longitudinal studies indicate that these compositional and functional changes can persist for months or even years, particularly after repeated or broad-spectrum therapies, and are influenced by the specific type of antibiotic and its administration method (169, 170). Post-antibiotic dysbiosis has been linked to increased risks of obesity, allergies, and autoimmune diseases when exposure occurs early in life, greater susceptibility to recurrent infections, alterations of the gut-brain axis affecting behavior and mood, and an increase in antimicrobial resistance genes within the intestinal ecosystem (171).

### 8.1 Prevention and mitigation strategies for antibiotic dysbiosis

The primary strategy is responsible antibiotic prescribing: selecting the most specific drug, limiting duration, avoiding unnecessary use for viral or self-limiting infections, and considering local alternatives (e.g., topical therapy). WHO and EMA guidelines emphasize antibiotic stewardship to minimize ecological impacts on the microbiota. Probiotics during or after antibiotic therapy can reduce antibiotic-associated diarrhea and limit opportunistic pathogen growth. Strains such as *Lactobacillus rhamnosus* GG and *Saccharomyces boulardii* reduce diarrhea risk by ~60% and may modulate inflammation and compete with *Proteobacteria*. New strains resistant to certain antibiotics and spore-forming bacteria allow concurrent use during therapy. Efficacy remains strain-specific, and probiotics alone do not fully restore biodiversity (172, 173).

Fermentable fibers, such as inulin, FOS, GOS, and mixed soluble fibers, promote SCFA-producing bacteria, reduce inflammation, and improve mucosal integrity, accelerating microbiota recovery. Diet is a powerful tool: diverse plant foods (>30 plants/week), legumes, whole grains, vegetables, fruits, and fermented foods (kefir, yogurt, miso, sauerkraut, kombucha) enhance microbial diversity, while limiting simple sugars and saturated fats reduces Proteobacteria overgrowth (174, 175).

Postbiotics, including butyrate, propionate, bacterial cell wall fractions, and organic acids, support post-antibiotic recovery by strengthening the intestinal barrier, reducing inflammation, and inhibiting pathogens such as *C. difficile*, even without live bacteria. They are safe for frail individuals and can be used during antibiotic therapy (176).

Fecal microbiota transplantation (FMT) is highly effective for recurrent *C. difficile* infections, restoring microbial biodiversity with >85% success. Its broader use for post-antibiotic dysbiosis is under investigation (177).

Species loss peaks within 72 h of antibiotics; partial recovery occurs within 2–4 weeks, with gradual reconstitution over 6–12 months. Nutritional and probiotic interventions in the first 2–6 weeks post-antibiotics are critical to maximize recovery (178).

## 9 Environmental supports for mothers unable to breastfeed

Maternal health is a fundamental determinant of infant wellbeing, especially when breastfeeding is not possible. Numerous scientific studies demonstrate that regular exposure to nature, defined as interaction with green spaces, sunlight, environmental biodiversity, and open environments, significantly contributes to reducing stress, modulating the inflammatory response, and improving the mother’s neuroendocrine regulation (179, 180). These effects have direct implications for the emotional and biological climate of the home environment, which in turn determines the maturation of the infant’s microbiota. Since chronic stress is associated with dysbiosis, intestinal permeability, and immune dysregulation, creating regular opportunities for outdoor wellbeing represents an indirect but biologically relevant intervention in supporting the entire family’s microbiota (181).

## 10 Healthcare professionals and educational innovations: economic implications of microbiota-based interventions

The perinatal period provides a critical window for proactive health interventions focused on prevention rather than treatment. Low-cost, non-invasive educational and nutritional strategies can effectively support maternal and infant health, with healthcare professionals playing a key role in guiding families on environmental, nutritional, and microbiological factors that shape microbial diversity (6, 182). Socioeconomic analyses suggest that targeted use of probiotics could generate substantial benefits for European healthcare systems. A Socio-Economic Impact Assessment, based on over 500 randomized trials involving 73,000 patients, estimated potential annual EU savings of more than €10 billion, accounting for healthcare costs and productivity losses. The analysis focused on seven common conditions: antibiotic-associated diarrhea, gut microbiota restoration, upper respiratory infections, lactose intolerance, oral health, and microbiota-gut-brain-mediated mental health. The largest savings were projected for antibiotic-associated diarrhea (€5.9–8.5 billion/year) and respiratory infections (€1.1–3.9 billion/year), with notable benefits also for lactose intolerance (€7–9.5 billion/year) and incremental gains for oral and mental health (IPA Europe 2025 policy report).

Even modest individual improvements, when applied across prevalent and recurrent conditions, can translate into significant macroeconomic gains. A targeted microbiota approach, including probiotics, prebiotics, and postbiotics, represents a cost-effective complement to conventional prevention policies, supporting the sustainability of healthcare systems (Table 2).

**Table 2:** The table summarizes the main determinants of microbial modulation.

Intervention strategy	Rationale/mechanisms	Main outcomes on microbiota and health	Level of evidence	Key references
Breastfeeding	HMOs, bioactive compounds, immune cells, maternal microbial transfer; bifidogenic effect	Reduced infections and allergy risk; improved immune and neurodevelopmental outcomes	High	(1, 10, 11, 31, 43, 48)
Modified infant formula	Partial functional mimicry of human milk (protein, lipids, DHA/ARA, MFGM)	Improved growth; modest cognitive and immune benefits	Moderate	(7, 34, 49, 54, 62)
Prebiotics (GOS, FOS, HMO-mimics)	Selective stimulation of Bifidobacterium and Lactobacillus; SCFA production	Bifidogenic effect; improved stool consistency; modest allergy prevention	Moderate	(73, 75, 76)
Probiotics (strain-specific)	Competitive exclusion of pathobionts; immune and barrier modulation	Reduced antibiotic-associated diarrhea; variable effects on colic and infections	Low–Moderate	(87, 172, 173)
Synbiotics	Synergistic prebiotic–probiotic interaction	Improved gut function; modest immune modulation	Low–Moderate	(92)
Postbiotics	Bioactive microbial metabolites or inactivated cells; no colonization required	Anti-inflammatory effects; improved mucosal resilience; high safety profile	Low	(86, 93, 94, 176)
Complementary feeding practices	Dietary diversity and fiber drive adult-like microbiota and SCFA production	Increased microbial diversity; metabolic and immune programming	Moderate–High	(12, 134, 136, 148, 149)

From this perspective, interventions based on diet, probiotics, prebiotics, and postbiotics represent a cost-effective complement to traditional preventive strategies, contributing to the sustainability of healthcare systems.

## 11 Future directions in public health for supporting non-breastfeeding families

Aggregate breastfeeding data often mask substantial inequalities linked to sociodemographic, economic, and cultural factors. Stratified analyses show higher initiation rates among non-Hispanic white, Hispanic, and particularly non-Hispanic Asian mothers, while non-Hispanic Black mothers consistently have lower rates (183). Public health targets, such as Healthy People 2020, were not met for this group in the 2018 birth cohort, reflecting persistent structural gaps.

Disparities also emerge across other social determinants, with lower breastfeeding rates among low-income, adolescent, and less-educated mothers, including WIC participants. These patterns reflect systemic barriers, including limited access to support services, unfavorable work conditions, reduced social protection, and fragmented perinatal care. Such inequalities contribute to health inequities, with reduced breastfeeding in non-Hispanic Black populations linked to higher risks of adverse infant outcomes, including acute otitis media, necrotizing enterocolitis, and infant mortality. Breastfeeding disparities thus serve as indicators of broader social and public health inequities.

Global health organizations (WHO, UNICEF, and PAHO) recognize that some families cannot breastfeed for medical, social, economic, or personal reasons. Policies must balance breastfeeding promotion with support for safe alternatives, such as donor human milk or regulated infant formula, to protect vulnerable infants while avoiding stigmatization. Early introduction of formula can affect gut microbiota and immune development, highlighting the importance of high-quality alternatives and proper feeding guidance.

International guidelines stress preventing inappropriate marketing of breast milk substitutes, ensuring equitable access to safe alternatives, and providing contextualized instructions for non-breastfeeding families, including hygienic formula preparation, appropriate feeding methods, and complementary feeding for 6–24 months. Supportive practices, skin-to-skin contact, kangaroo care, and close feeding promote maternal–infant bonding, neuroendocrine regulation, and beneficial microbial transmission even in formula-fed infants.

Integrated, consistent care in health facilities, exemplified by the Baby-Friendly Hospital Initiative (BFHI), is essential. Written protocols, healthcare worker training, and nonjudgmental support for all feeding modalities ensure equity, protect infant health, and promote optimal microbial and immune development.

## 12 Conclusion

Early-life microbiota health should be recognized as a public health priority that extends beyond breastfeeding. While human milk remains a cornerstone of infant nutrition, current evidence demands a paradigm shift: microbial, immune, and metabolic programming are shaped by a network of environmental, nutritional, and relational determinants, many of which are amenable to policy intervention.

Health systems that focus narrowly on breastfeeding rates risk overlooking critical opportunities for prevention. Protecting infant health requires integrated strategies that ensure microbiota-supportive nutrition, safe environmental exposures, responsible antibiotic use, relational continuity within the mother–infant dyad, and equitable access to evidence-based guidance during the first 1,000 days of life. These determinants are not marginal; they are central drivers of long-term population health and health equity.

The One Health Decalogue provides a pragmatic framework to operationalize this shift. By translating microbiome science into clear, actionable principles, it offers a tool to bridge biological vulnerabilities, clinical uncertainty, and policy fragmentation, particularly for families who cannot breastfeed. Embedding such an approach into maternal–child health policies has the potential to reduce preventable disease, counter social inequities, and redefine early-life prevention in a scientifically grounded and socially inclusive manner. Moving beyond a milk-centered narrative is not about diminishing breastfeeding, but about expanding responsibility, from individual mothers to healthcare systems, environments, and public institutions.

This transition is essential to align early-life care with contemporary microbiome science and with the goals of sustainable, equitable public health (Table 3).

**Table 3:** Future directions.

Policy theme	Implications/Actions
Reframe early-life prevention	Apply One Health perspective: nutrition, environment, caregiving Move beyond exclusive breastfeeding focus
Protect non-breastfed infants	Provide structured, evidence-based support Ensure equity and continuity of care
Leverage low-cost, high-impact interventions	Skin-to-skin contact Exposure to biodiverse environments Balanced hygiene & microbiota-informed complementary feeding Integrate into maternal–child health programs
Bridge science and policy	Use One Health Decalogue to translate research into practice Develop clinical guidelines and public health strategies
